# Social determinants affecting the use of complementary and alternative medicine in Japan: An analysis using the conceptual framework of social determinants of health

**DOI:** 10.1371/journal.pone.0200578

**Published:** 2018-07-16

**Authors:** Jimpei Misawa, Rie Ichikawa, Akiko Shibuya, Yukihiro Maeda, Teruyoshi Hishiki, Yoshiaki Kondo

**Affiliations:** 1 Department of Health Care Services Management, Nihon University School of Medicine, Itabashi, Tokyo, Japan; 2 Department of Pediatrics and Child Health, Nihon University School of Medicine, Itabashi, Tokyo, Japan; 3 Department of Information Science, Faculty of Science, Toho University, Funabashi, Chiba, Japan; Instituto Nacional de Medicina Genomica, MEXICO

## Abstract

This study aims to use the conceptual framework of social determinants of health (SDH) to elucidate the social determinants that affect the use of complementary and alternative medicine (CAM) from the perspectives of both intermediary and structural determinants. Data were derived from a survey mailed to 1,500 randomly selected residents (20–69 years old; May–July 2009) of Sendai city in Japan. A generalized linear model was used in the analysis, with CAM use over the past one month as the dependent variable, SDH structural and intermediary determinants as independent variables, and demographic characteristics, indicators of health status, and the evaluation of health or healthcare systems as control variables. The prevalence of CAM usage was 62.1%. The generalized linear model showed that middle subjective social status (OR = 1.47; 95% CI: 1.04–2.07) as structural determinants was significantly associated with CAM usage. Adding the intermediary determinants, the same effect was observed. When demographic characteristics, indicators of health status, and the evaluation of health or healthcare systems were introduced as control variables, the associations of the structural determinants disappeared, revealing that hope (OR = 1.25; 95%CI: 1.04–1.50) as intermediary determinants was associated with the use of CAM. Female sex (OR = 1.47; 95% CI: 1.02–2.12) and health anxiety (OR = 1.68; 95% CI: 1.20–2.34) were associated with CAM usage. We found that intermediary rather than structural determinants were associated with CAM usage. Hope as an intermediary determinant was particularly associated with CAM usage.

## Introduction

With growing health awareness and the increased popularity of self-management of health, interest in Complementary and Alternative Medicine (CAM) is growing [1]. The expert panel of the National Center for Complementary and Integrative Health (NCCIH) defines CAM as follows: “Complementary and alternative medicine (CAM) is a broad domain of resources that encompasses health systems, modalities, and practices and their accompanying theories and beliefs, other than those intrinsic to the dominant health system of a particular society or culture in a given historical period. CAM includes such resources perceived by their users as associated with positive health outcomes. Boundaries within CAM and between the CAM domain and the domain of the dominant system are not always sharp or fixed” [2]. In particular, CAM can be classified into “Natural Products” such as dietary supplements, herbs, and probiotics; and “Mind and Body Practices” such as yoga, chiropractic and osteopathic manipulation, meditation, and massage therapy [3].

Review articles have reported that CAM usage prevalence rates among adults in some developed countries between 5% and 76% [4, 5]. Furthermore, the prevalence of CAM usage in EU countries varied widely (0.3% to 86%) [6]. In particular, according to the report published by the Centers for Disease Control and Prevention's (CDC) National Center for Health Statistics (NCHS) on CAM usage in the past one year, the proportion of persons using some form of CAM was 32.3% in 2002, 35.5% in 2007, and 33.2% in 2012, showing a high prevalence of CAM usage in the US (based on age-adjusted data) [7]. Thus, the global prevalence of CAM usage is very high.

The usage status of CAM is similar in Japan. The proportions of CAM users in cancer patients, patients with chronic diseases, and outpatients in family medicine clinics were 44.6%, 75.1%, and 80.0%, respectively [8–10]. Moreover, according to a national telephone survey conducted in Japan, which was not restricted to patients only, the proportion was 76% [11]. It has also been reported that the proportion was 51% in factory workers [12], and 60% in residents of regional municipalities [13, 14]. Compared with other countries, CAM usage in the general Japanese population appears to be significantly high.

According to a review report on factors related to CAM usage during 1995 to 2006 [15], CAM usage is related to female sex, high educational level, middle age, and health issues. However, when analyzing factors related to CAM usage, we need to consider not only demographic characteristics and the evaluation of healthcare systems, but also factors based on the social context in which humans live as described in the definition of CAM given above. Therefore, there is room for analysis of CAM usage factors based on social determinants of health (SDH). The definition of SDH states, "The social determinants of health (SDH) are the conditions in which people are born, grow, work, live, and age, and the wider set of forces and systems shaping the conditions of daily life. These forces and systems include economic policies and systems, development agendas, social norms, social policies, and political systems” [16]. In the framework regarding the relationship between SDH and health/well-being, it has been reported that not only do structural determinants such as social system and socioeconomic position (being high or low in social status) determine health/well-being status, but the status of intermediary determinants such as material circumstances, behaviors, biological factors, and psychosocial factors, derived from the structural determinants, also affect health/well-being status [17].

The US report mentioned above reported that CAM usage is related to race/ethnicity [7]. Another review of CAM usage revealed that CAM usage is also related to educational level [18]. Although these reports show factors related to CAM usage from the SDH perspective, they only explain the association between structural determinants and CAM usage. The issue here is that composite associations between structural determinants, intermediary determinants, and CAM usage have not been taken into account. Moreover, the relationship between the social determinants of health and CAM usage in Japan has not yet been elucidated.

Therefore, the study aims to elucidate the social determinants that affect the CAM usage from both intermediary and structural determinants perspectives within the conceptual framework of social determinants of health, using social survey data from local Japanese residents.

## Methods

### Data

The data was derived from the "Health and Lifestyle Survey" conducted during May through July 2009. The mail survey was conducted among 1,500 randomly selected male and female residents of Sendai City, aged 20 to 69 years. Sendai is a city located approximately 300 km north of Tokyo. The survey received 1,018 responses (a response rate: 68.6%). This study was approved by the Tohoku University Medical Sciences' ethical review board (No. 2009–33, April 27, 2009). We interpreted the voluntary return of the self-administered questionnaire in the postal survey as informed consent. The data were analyzed anonymously. The data are available from a supporting information file (S1 Dataset). The questionnaire of this survey is available in a supporting information file (S1 Appendix).

### Variables

CAM usage was the dependent variable and was measured as a dichotomous variable (use/not use CAM). In this study, we defined CAM as therapies or products consisting of supplements, nutritional /nourishing drinks, massage, health promoting tools, Chinese (kampo) medicine, chiropractic therapy, aromatherapy, acupuncture, and qi gong. Participants were asked if they had ever used any of the CAM therapies or products listed above. Since we aimed to clarify the social determinants affecting the use of CAM in general, we employed a simple variable of whether CAM was used rather than variables such as type of CAM and frequency of CAM usage. Additionally, in general, it is common to ask about usage over the past one year, but in the present study, only usage over the past one month was studied. Even if participants had used CAM only once in the past year, they would be classified as “have used CAM.” We thought that this did not realistically indicate the actual use of CAM. We considered it appropriate to measure CAM usage in the past month to accurately grasp the practical situation of using CAM. Therefore, we investigated CAM usage over the past one month in the survey.

The independent variables were socioeconomic status as the structural determinants of SDH. With respect to socioeconomic status, four variables of subjective social status, educational level, occupation, and equivalent household income were used. We used subjective social status as a subjective indicator of one’s own social status, and educational level, occupation, and equivalent income as objective indicators of one’s own social status. With respect to subjective social status, participants were asked "Assuming that the present society in Japan is classified into the following five classes, where do you think you belong?" and then asked to select one of the following five positions: top, top of the middle, bottom of the middle, top of the bottom, and bottom of the bottom. As Japanese people tend to recognize their own status compared with others, subjective evaluation is an effective measure of socioeconomic status [19]. We arranged into three categories of high (containing top and top of the middle), middle (bottom of the middle), and low (containing top of the bottom and bottom of the bottom) for analysis purposes since few respondents were in the “top” and “bottom of the bottom” stratum categories. This classification was conducted with reference to previous research [20, 21]. The educational level was classified into three categories of high school graduation, vocational school or junior college graduation, and university graduation. Occupation was classified into five categories of regular employment, non-regular employment, self-employed or freelance, unemployed, and housework. This question and classification was conducted with reference to previous research [22]. Equivalent household income was calculated by dividing the annual household income by the square root of the household size and then classified into 4 categories of less than 2,000,000 yen, 2,000,000–4,000,000 yen, above 4,000,000 yen, and no answer. The category of "no answer" was added here because a large proportion of the participants did not respond to the income related questions. It has been reported that common characteristics of persons who do not respond to income related questions are elderly or young, low education level, and unemployed [23, 24]. In addition, “no answer” is typically used as a proxy indicator of low socioeconomic status in research studies of social inequalities in health [25]. In view of this, instead of dealing with the no response group uniformly as missing data and excluding it from the analysis, the "no answer" category was created and included in the analysis as it was deemed rational to consider this as a proxy indicator of low socioeconomic position.

Regarding the intermediary determinants of SDH, in this study we used psychosocial factors of economic anxiety, hope, and life satisfaction. With respect to economic anxiety, the question was "Do you have any anxiety about your future income and assets?" and responses were obtained in a four-point scale of highly anxious, somewhat anxious, not so anxious, and not at all anxious. In the analysis, these responses were grouped into two values of "anxious" (containing the highly anxious and somewhat anxious responses) and "not anxious" (containing the not so anxious and not at all anxious responses) for the analytical purpose of simple understanding of whether participants were anxious or not. This question was made with reference to previous research [26]. With respect to hope, the question was "Do you have hope for your life in the future?" and responses were obtained in a five-point scale of high hope (= 5), hope (= 4), not sure (= 3), not much hope (= 2), and no hope at all (= 1). This question was made with reference to previous research [27].With respect to life satisfaction, the question was "Are you currently satisfied with your life in general?", and the responses were obtained in a five-point scale of satisfied (= 5), partly satisfied (= 4), not sure (= 3), partly dissatisfied (= 2), and dissatisfied (= 1). In the analysis, we treated hope and life satisfaction as continuous variables, because hope did not have a validated cut-off point and, if treated as an ordinal variable, may have reduced the information obtained from the data. Moreover, life satisfaction is typically treated as a continuous variable in the social science, including sociology and economics [28, 29]. Thus, to ensure comparability with other studies, we also treated life satisfaction as a continuous variable.

Regarding the control variables, we used the demographic characteristics of sex, age, and marital status. In addition, we employed indicators of health status of health-related quality of life (QOL), self-rated health, and chronic disease. Moreover, we adopted health anxiety and healthcare satisfaction as the evaluation of health or healthcare systems. With respect to age, categories of 20 to 29 years, 30 to 39 years, 40 to 49 years, 50 to 59 years, and 60 to 69 years were used. Marital status was categorized into married, unmarried, and divorced/widowed. Previous research has indicated an association between health and marital status, particularly in men who were divorced/widowed or unmarried in Japan [30]. Therefore, we employed the marital statuses of married, unmarried, and divorced/widowed. With respect to health-related QOL, the Japanese version of the SF-8 was used [31]. The SF-8 is a comprehensive health-related QOL instrument used in subjective evaluations of health status. It constitutes eight health profile dimensions of general health (GH), physical functioning (PF), role physical (RP, role limitations because of physical health), bodily pain (BP), vitality (VT), social functioning (SF), mental health (MH), and role emotional (RE, role limitations because of emotional problems). In the analysis, the scores of the eight dimensions were summarized into two factors and their summary scores were used in the analysis. The two summary scores used were PCS (Physical Component Summary) and MCS (Mental Component Summary). With respect to self-rated health, the question asked was "How do you rate your own health in general?", and the responses were obtained in a five-point scale of excellent, very good, good, fair, and poor. Fair/poor health has been indicative of the presence of health distress and/or disease and increases mortality risk [32]. For this reason and following existing practice [33–35], in the analysis, data were classified into two categories of "good" (containing the excellent, very good, and good responses) and "bad" (containing fair and poor responses). Chronic illness was categorized into none and illness. Since this study was conducted by mail survey, if participants did not accurately understand their own chronic disease, an incorrect disease could be marked. Thus, participants were simply asked whether they currently had any chronic illnesses, and we measured chronic disease with yes/no. With respect to health anxiety, the question was "Do you currently have any anxiety about your health?", and responses were obtained in a four-point scale of always anxious, sometimes anxious, not so anxious, and not at all anxious. In the analysis, these were regrouped into two values of "anxious" (containing the always anxious and sometimes anxious responses) and "not anxious" (containing the not so anxious and not at all anxious responses) for the analytical purpose of a simple understanding of whether participants were anxious or not. With respect to healthcare satisfaction, the question was "Are you satisfied with the current healthcare system in general?", and responses were obtained in a four-point scale of highly satisfied, satisfied, partly dissatisfied, and dissatisfied. In the analysis, these were regrouped into two values of "satisfied" (containing the highly satisfied and satisfied responses) and "dissatisfied" (containing the partly dissatisfied and dissatisfied responses) for the analytical purpose of a simple understanding of whether participants were satisfied or not.

### Analysis method

CAM usage (presence of CAM usage = 1) was examined as a dichotomized outcome variable within a generalized linear model (Logit link function). First, model 1, wherein the socioeconomic status was incorporated as structural determinant of SDH, was analyzed. Next, model 2, wherein economic anxiety, hope, and life satisfaction were incorporated as intermediary determinants of SDH, was analyzed. Thereafter, model 3, in which demographic characteristics were added, model 4, in which health-related QOL, self-rated health, and chronic disease were added, and model 5, in which health anxiety and healthcare satisfaction were added, were analyzed. In the analysis, excluding the participants who did not respond to the questions regarding the principal variables, the sample size used was 838. The analysis was performed using R 3.4.1 [36], with a significance level of 5%.

## Results

### Prevalence of CAM usage

The proportion of participants who had used CAM in the past one month was 62.1% (Table 1). The proportions of participants using supplements (59.2%) and nutritional /nourishing drinks (49.4%) were high.

**Table 1:** The prevalence of CAM usage over the past one month.

	n	%
Not Use	318	37.9
Use	520	62.1
Details (Multiple answer) (n = 520)		
Supplements	308	59.2
Nutritional /nourishing drinks	257	49.4
Massage	75	14.4
Health promoting tool	55	10.6
Chinese (Kampo) medicine	49	9.4
Chiropractic therapy	48	9.2
Aromatherapy	18	3.5
Acupuncture	17	3.3
Qi gong	1	0.2
Other	10	1.9

### Structural and intermediary determinants of SDH

With respect to SDH structural determinants (Table 2), for equivalent household income the largest proportion of participants (38.8%) was from the category of between 2,000,000 and 4,000,000 yen, for educational level the largest proportion was from the completed high school category (46.2%), for occupation more than 40% were from the regular employment category, and for subjective social status the largest proportion was from the middle position category.

**Table 2:** Social determinants of health.

	n	%	Mean	Standard Deviation
Structural determinants				
Equivalent household income				
less than 2,000,000 yen	155	18.5		
2,000,000–4,000,000 yen	325	38.8		
Above 4,000,000 yen	248	29.6		
No answer	110	13.1		
Educational levels				
High school	387	46.2		
Vocational school or junior college	217	25.9		
University	234	27.9		
Occupation				
Regular employment	350	41.8		
Non-regular employment	204	24.3		
Self-employed or freelance	50	6.0		
Unemployed	103	12.3		
Housework	131	15.6		
Subjective social status				
Low	251	30.0		
Middle	406	48.4		
High	181	21.6		
Intermediary determinants				
Economic anxiety				
Not anxious	127	15.2		
Anxious	711	84.8		
Hope			3.0	1.0
Life satisfaction			3.3	1.1

With respect to the intermediary determinants, more than 80% of the participants were in the "anxious" category of economic anxiety. For hope and life satisfaction the mean values were 3.0±1.0 and 3.3±1.1, respectively.

### Demographic characteristics, health status indicators, and evaluation of health or healthcare system

With respect to the demographic characteristics (Table 3), mean age was 46.4 (±13.2) years, and 54.1% were females (Table 3). With respect to the SF-8 showing health status, the mean values for PCS and MCS were 48.6±6.9 and 46.8±7.7, respectively. With respect to the self-rated health, more than 80% were from the "good" category. Approximately 40% of the participants have chronic disease. It was observed that approximately 70% were in the "anxious" category regarding health. Approximately 60% were in the "dissatisfied" category regarding healthcare system.

**Table 3:** Control variables.

	n	%	Mean	Standard Deviation
Demographic characteristics				
Gender				
Male	385	45.9		
Female	453	54.1		
Age categories			46.4	13.7
20 to 29 years old	107	12.8		
30 to 39 years old	176	21.0		
40 to 49 years old	190	22.7		
50 to 59 years old	188	22.4		
60 to 69 years old	177	21.1		
Marital status				
Married	565	67.4		
Unmarried	200	23.9		
Divorced/widowed	73	8.7		
Indicators of health status				
PCS		48.6	6.9	
MCS		46.8	7.7	
Self-rated health				
Bad	135	16.1		
Good	703	83.9		
Chronic disease				
None	526	62.8		
Illness	312	37.2		
The evaluation of health or healthcare systems				
Health anxiety				
Not anxious	258	30.8		
Anxious	580	69.2		
Healthcare satisfaction				
Dissatisfied	498	59.4		
Satisfied	340	40.6		

### Results of generalized linear models with CAM usage as the dependent variable

Table 4 shows the results of the generalized linear models. In model 1, the model using the SDH structural determinants showed clearly that CAM usage was associated with middle subjective social status (OR = 1.47, 95% CI: 1.04–2.07). In model 2 where the intermediary determinants were added, similar to model 1, an association between CAM usage and middle subjective social status was observed (OR = 1.57, 95% CI: 1.09–2.26). In model 3 where demographic characteristics were added, associations between CAM usage and female sex (OR = 1.57, 95% CI: 1.09–2.25), and the age category of 40 to 49 years (OR = 1.80, 95% CI: 1.01–3.19) were observed. In model 4 where health-related QOL, self-rated health, and chronic disease were added, associations similar to model 3 for sex (OR = 1.48, 95% CI: 1.03–2.15) and age categories of 40 to 49 years (OR = 1.85, 95% CI: 1.03–3.32) were observed. Additionally, negative associations were observed for the health-related QOL factors of PCS (OR = 0.96, 95% CI: 0.93–0.98) and MCS (OR = 0.97, 95% CI: 0.94–0.99). For the SDH intermediary determinant of hope (OR = 1.24, 95% CI: 1.04–1.50) an association was observed. Finally, in model 5 where health anxiety and healthcare satisfaction were added, associations for sex (OR = 1.47, 95% CI: 1.02–2.12), health-related QOL factors of PCS (OR = 0.96, 95% CI: 0.94–0.99) and MCS (OR = 0.97, 95% CI: 0.95–0.99), and hope (OR = 1.25, 95% CI: 1.04–1.50) were consistently observed, but the association for age disappeared. In contrast, an association with health anxiety (OR = 1.68, 95% CI: 1.20–2.34) was observed.

**Table 4:** The generalized linear model (link function: Logit) with CAM usage.

	Model 1			Model 2			Model 3			Model 4			Model 5		
	OR	(95% CI)	P value	OR	(95% CI)	P value	OR	(95% CI)	P value	OR	(95% CI)	P value	OR	(95% CI)	P value
Income															
Less than two million yen	1.00			1.00			1.00			1.00			1.00		
200 to four million yen	1.09	(0.72–1.64)	.691	1.11	(0.73–1.69)	.616	1.06	(0.69–1.62)	.784	1.07	(0.69–1.64)	.763	1.10	(0.71–1.69)	.679
Above four million yen	1.38	(0.85–2.23)	.193	1.42	(0.87–2.30)	.159	1.21	(0.74–2.00)	.445	1.22	(0.74–2.03)	.434	1.29	(0.78–2.16)	.325
No answer	1.05	(0.63–1.77)	.843	1.05	(0.62–1.77)	.857	1.03	(0.60–1.75)	.924	1.11	(0.65–1.91)	.693	1.21	(0.70–2.09)	.495
Educational levels															
High school	1.00			1.00			1.00			1.00			1.00		
Vocational school or junior college	1.12	(0.79–1.60)	.520	1.11	(0.78–1.58)	.569	1.09	(0.76–1.58)	.642	1.06	(0.73–1.55)	.748	1.09	(0.74–1.59)	.669
University	0.87	(0.61–1.24)	.447	0.88	(0.61–1.26)	.478	0.93	(0.64–1.34)	.697	0.92	(0.63–1.33)	.651	0.92	(0.63–1.35)	.676
Occupation															
Regular employment	1.00			1.00			1.00			1.00			1.00		
Non-regular employment	1.29	(0.89–1.88)	.187	1.34	(0.92–1.96)	.133	1.10	(0.72–1.67)	.659	1.11	(0.73–1.71)	.620	1.12	(0.73–1.71)	.615
Self-employed or freelance	1.07	(0.58–2.02)	.829	1.07	(0.58–2.02)	.835	0.94	(0.49–1.81)	.839	0.94	(0.49–1.83)	.845	0.92	(0.48–1.82)	.817
Unemployed	1.37	(0.85–2.24)	.201	1.43	(0.88–2.34)	.153	1.43	(0.85–2.44)	.183	1.36	(0.80–2.34)	.254	1.29	(0.76–2.22)	.354
Housework	0.80	(0.52–1.22)	.296	0.83	(0.55–1.28)	.403	0.61	(0.36–1.01)	.057	0.60	(0.36–1.02)	.058.	0.61	(0.36–1.03)	.062
Subjective social status															
Low	1.00			1.00			1.00			1.00			1.00		
Middle	1.47	(1.04–2.07)	.028	1.57	(1.09–2.26)	.016	1.43	(0.99–2.08)	.060	1.44	(0.98–2.10)	.061	1.44	(0.98–2.11)	.062
High	1.16	(0.74–1.82)	.514	1.24	(0.75–2.06)	.407	1.12	(0.67–1.89)	.662	1.13	(0.67–1.91)	.654	1.13	(0.67–1.93)	.645
Economic anxiety															
No anxious				1.00			1.00			1.00			1.00		
Anxious				1.01	(0.65–1.57)	.948	1.01	(0.65–1.57)	.954	1.01	(0.64–1.58)	.963	1.00	(0.63–1.56)	.993
Hope				1.13	(0.95–1.34)	.159	1.17	(0.98–1.40)	.090	1.24	(1.04–1.50)	.021	1.25	(1.04–1.50)	.020
Life satisfaction				0.86	(0.74–1.00)	.051	0.86	(0.73–1.00)	.054	0.94	(0.80–1.11)	.487	0.95	(0.80–1.13)	.553
Gender															
Male							1.00			1.00			1.00		
Female							1.57	(1.09–2.25)	.015	1.48	(1.03–2.15)	.035	1.47	(1.02–2.12)	.042
Age categories															
20 to 29 years old							1.00			1.00			1.00		
30 to 39 years old							0.99	(0.58–1.69)	.968	0.99	(0.58–1.70)	.974	1.00	(0.58–1.72)	.991
40 to 49 years old							1.80	(1.01–3.19)	.045	1.85	(1.03–3.32)	.040	1.77	(0.98–3.20)	.057
50 to 59 years old							1.45	(0.80–2.64)	.219	1.41	(0.77–2.61)	.269	1.35	(0.73–2.51)	.337
60 to 69 years old							1.15	(0.61–2.14)	.670	1.18	(0.61–2.28)	.621	1.11	(0.57–2.16)	.749
Marital status															
Married							1.00			1.00			1.00		
Unmarried							0.89	(0.58–1.37)	.580	0.93	(0.6–1.44)	.733	0.95	(0.61–1.47)	.802
Divorced/widowed							0.93	(0.54–1.63)	.796	0.97	(0.56–1.71)	.907	0.99	(0.57–1.76)	.977
PCS										0.96	(0.93–0.98)	<.001	0.96	(0.94–0.99)	.003
MCS										0.97	(0.94–0.99)	.003	0.97	(0.95–0.99)	.014
Self-rated health															
Bad										1.00			1.00		
Good										1.04	(0.63–1.69)	.879	1.12	(0.68–1.83)	.665
Chronic disease															
No										1.00			1.00		
Illness										1.02	(0.72–1.44)	.906	0.99	(0.70–1.41)	.961
Health anxiety															
No anxious													1.00		
Anxious													1.68	(1.20–2.34)	.002
Healthcare satisfaction															
Dissatisfied													1.00		
Satisfied													0.98	(0.72–1.33)	.903
n	838			838			838			838			838		
Nagelkerke R^2^	0.02			0.03			0.06			0.09			0.10		
-2×log likelihood	1098.5			1093.9			1077.5			1056.3			1047.0		

## Discussion

The study aimed to elucidate the social determinants that affect the use of CAM from both intermediary and structural determinant perspectives in the conceptual framework of SDH, by analyzing social survey data from residents of Sendai, Japan. The response rate was 68.6%, which was a fairly good response. Since this survey was postal rather than interview-based, we do not know why the remaining 41.4% of respondents did not answer. In general, however, non-response does not seriously bias analyses of disparities in social determinants in health status [37]. Therefore, since it is unlikely that non-respondents did not respond in relation to SDH or CAM usage, we believe that distribution of respondents was not biased and that the results of this study are valid.

The survey showed that the proportion of CAM usage was approximately 60%. Compared to a report that cited 76% of Japanese having used CAM [11], it appears the prevalence of CAM in the survey is low. The result would be related to be limited to usage over the past one month. However, compared to a report that around 30% of American having used CAM [7], the prevalence of CAM usage among Japanese people is high.

The results of the generalized linear model (using link function logit) with CAM usage as the dependent variable in model 1 using the SDH structural determinants only, suggest that participants with middle subjective social status are more likely to use CAM compared to those with the lower. Similar results were found when intermediary determinants were included in the analysis. However, when demographic characteristics, indicators of health status, and the evaluation of health or healthcare systems were introduced as control variables, the associations of the structural determinants disappeared. Instead, it was found that the more hope that people have the more they use CAM, which shows the effects of intermediary determinants.

Previous studies have reported that female sex, high educational level, middle aged, and health issues are some of the factors that influence usage of CAM [15]. In this study, it was also observed that females use CAM more than males, and the higher the health-related QOL score in both physical and mental components the lower the use of CAM, thus supporting findings from the previous studies [6, 15].

In model 3 and 4, an association with the age category of 40 to 49 years was observed, supporting findings from previous studies [15, 38]. However, after introducing health anxiety and healthcare satisfaction in model 5, an association with health anxiety was observed but the association with age disappeared and also the odds ratio (OR) was reduced. We think that this may likely be due to the fact that in the middle age group of 40 to 49 years, CAM usage was associated with health anxiety. This suggests that health anxiety potentially acts as a confounding factor between middle age and CAM usage. Moreover, in our study, there was no direct association between middle age and CAM usage. For the other variables, even after introducing the variables regarding the evaluation of health or healthcare systems, there were no significant changes in the degree of the respective associations. Thus, we believe that there likely was a strong association between age and health anxiety. Therefore, in the future, in order for middle-aged people to properly use CAM, it is important to develop a healthcare system and a social system to prevent middle-aged people from feeling anxious about their own health.

A significant difference from the findings of previous research observed in the present study was regarding the determinants related to SDH. Hitherto, it had been observed that objective socioeconomic status indicators including educational and income levels were the structural determinants associated with CAM usage [4, 15, 39, 40]. However, the results of the present study suggest that the structural determinant of subjective social status as a structural determinant of SDH is associated with CAM usage. With regard to the effects of subjective and objective socioeconomic status on health-related indicators, a previous study has reported that objective socioeconomic status indicators such as income and educational levels had direct and indirect effects on health-related indicators [41]. However, in the study, objective socioeconomic status had no effect on CAM usage, only subjective social status had an effect on CAM usage. Therefore, we found that subjective social status, rather than educational and income level, was robustly associated with CAM usage, and thus may be related to a strong consciousness of social standing/status among the Japanese, because previous study has reported that the Japanese are conscious about relative comparisons of oneself with others [19]. Moreover, since subjective social status better explains subjective health evaluations [42], we believe that it may be likely that a similar tendency applies with respect to subjective decisions such as CAM usage in our study. Thus, subjective social hierarchy, such as subjective social status, rather than objective social hierarchy, such as educational level, is likely a more relevant structural determinant among Japanese people.

However, in model 3 and the ones after, when demographic characteristics, indicators of health status, and the evaluation of health or healthcare systems were introduced, the odds ratio for subjective social status reduced significantly, obliterating the association. Additionally, in model 4 and the ones after, instead of the structural determinants, the intermediary determinants show associations with CAM usage. Thus, although previous researches have shown that structural determinants, such as educational and income level, are associated with CAM usage [4, 15, 39, 40], our study indicates that intermediary determinants are associated with CAM usage. We believe that this is likely due to the unique characteristics of the Japanese society, such as less social inequality than other countries [43]. We believe that since the level of social inequality in Japan is low, structural determinants were less likely to influence CAM usage. In fact, as noted in a report regarding a study on the Gini coefficient in the OECD countries during 1960 to 2008, social inequality has been observed to be low in Japan [43]. In view of the fact that differences in social status are difficult to find in Japan, it is difficult to explain CAM usage through structural determinants, and we conclude that intermediary determinants, which are influenced by the structural determinants, more directly influence people’s psychosocial functioning, and were more likely to be associated with CAM usage. Thus, for future research, examining social structural differences, such as social context, are important for clarifying the factors of CAM usage.

Next, we consider the significance of the association of hope (as an intermediary determinant) with CAM usage. The association of hope with CAM usage was observed in model 4 and the later ones that included the variable regarding indicators of health status and the evaluation of health or healthcare systems. This means that this result may be due to the likely association between health consciousness and hope. In fact, having a purpose in life leads to greater longevity [44]. Moreover, according to hope theory [45], “hope reflects individuals’ perceptions regarding their capacities to clearly conceptualize goals, develop the specific strategies to reach those goals (pathways thinking), and initiate and sustain the motivation for using those strategies (agency thinking).” Thus, hope is goal-directed perceived capacity, and is associated with better health in the future. Therefore, the association between hope as an intermediary determinant and CAM usage as observed in the present study can also likely be interpreted as a goal-directed investment in the future self that is especially associated with good personal health.

Lastly, we describe the limitations of the present study and future challenges. In the present study, we only focused on the use of CAM in general. Therefore, we believe there is room for studies to be conducted on various types of therapies encompassed by CAM. In reality, even though it was observed in the present study that the use of nutritional/nourishing drinks is quite high, in a study by National Center for Complementary and Integrative Health where similar observations regarding the use of supplements were made, it was also found that the use of "Mind and Body Practices" such as yoga, tai chi, and qigong [7] was also high. Thus, we believe that more detailed studies on factors affecting CAM usage are necessary. It is particularly necessary to examine the relationship between specific therapy use and hope, and to consider meaningful actions and policies for using such therapies. Moreover, we believe that it is necessary to clarify the relationship with CAM usage considering the influence of various chronic diseases for future research. In the present study based on the conceptual framework of SDH, although we elucidated the association between SDH and CAM usage, in particular, we used structural determinant at individual level such as socioeconomic status, and psychosocial factors as intermediary determinants in the analysis. As the dataset included missing values, we should be careful while interpreting missing value groups. In addition, since structural determinants include macro-economic elements such as social policy, and intermediary determinants include physical environments such as housing conditions or health-related activities [46], we believe that further studies including these concepts are required. In particular, analyzing data from a macro perspective based on social structure is likely to be useful in planning social and health policies that promote appropriate CAM usage. We need to clarify the effect of social structure on CAM usage, taking into consideration the impact of hope. Moreover, since the survey was conducted, healthcare programs such as preventive long-term care for the elderly have undergone major changes. Since the development of these programs might be related to intermediary determinants of SDH, an analysis that considers the influence of changes in health care delivery will be necessary. Finally, the surveyed subjects were local residents in Japan. Therefore, it is difficult to amplify the results to the general Japanese population. However, we were able to clarify the social determinants of CAM usage from the perspective of the SDH framework. We observed that intermediary rather than structural determinants were associated with CAM usage. Hope was a particularly important intermediary determinant associated with CAM usage. This will contribute to social policy and action for future CAM usage in Japan. By implementing further investigations on the relationship between hope and CAM usage using a big data approach, researchers will be able to establish appropriate Japanese social policies that will contribute to prompt appropriate use of CAM in the future.

## Supporting information

S1 Dataset Social survey data. (XLSX)

S1 Appendix Questionnaire. (DOCX)
