# Supplementary material for: Social determinants affecting the use of complementary and alternative medicine in Japan: An analysis using the conceptual framework of social determinants of health
Source: PLoS One. 2018 Jul 16;13(7):e0200578. doi: 10.1371/journal.pone.0200578 (PMC6047791; doi:10.1371/journal.pone.0200578)
Supplement: S1 Appendix — (DOCX) [file pone.0200578.s002.docx]

| Health and Lifestyle Survey |
| --- |

Q1.1. Gender

1. Male
2. Female

Q1.2. Please enter your age on your last birthday. (Enter the specific numbers.)

| Your age on your last birthday |  | years old |
| --- | --- | --- |

Q2. How do you rate your own health in general?

1. Excellent
2. Very good
3. Good
4. Fair
5. Poor

Q3. Do you currently have any anxiety about your health?

1. Anxious
2. Somewhat anxious
3. Not so anxious
4. Not at all anxious

Q4. Do you currently have any chronic illnesses?

1. Yes
2. No

Q5.1. Overall, how would you rate your health during the past 4 weeks?

1. Excellent
2. Very Good
3. Good
4. Fair
5. Poor
6. Very Poor

Q5.2. During the past 4 weeks, how much did physical health problems limit your physical activities (such as walking or climbing stairs)?

1. Not at all
2. Very little
3. Somewhat
4. Quite a lot
5. Could not do physical activities

Q5.3. During the past 4 weeks, how much difficulty did you have doing your daily work, both at home and away from home, because of your physical health?

1. Not at all
2. Very little
3. Somewhat
4. Quite a lot
5. Could not do daily work

Q5.4. How much bodily pain have you had during the past 4 weeks?

1. None
2. Very mild
3. Mild
4. Moderate Severe
5. Very severe

Q5.5. During the past 4 weeks, how much energy did you have?

1. Very much
2. Quite a lot
3. Some
4. A little
5. None

Q5.6. During the past 4 weeks, how much did your physical health or emotional problems limit your usual social activities with family or friends?

1. Not at all
2. Very little
3. Somewhat
4. Quite a lot
5. Could not do social activities

Q5.7. During the past 4 weeks, how much have you been bothered by emotional problems (such as feeling anxious, depressed or irritable)?

1. Not at all
2. Slightly
3. Moderately
4. Quite a lot
5. Extremely

Q5.8. During the past 4 weeks, how much did personal or emotional problems keep you from doing your usual work, school or other daily activities?

1. Not at all
2. Very little
3. Somewhat
4. Quite a lot
5. Could not do daily activities

Q7. Are you currently satisfied with your life in general?

1. Satisfied
2. Partly satisfied
3. Not sure
4. Partly dissatisfied
5. Dissatisfied

Q8. Do you have hope for your life in the future?

1. High hope
2. Hope
3. Not sure
4. Not much hope
5. No hope at all

Q9. Do you have any anxiety about your future income and assets?

1. Highly anxious
2. Somewhat anxious
3. Not so anxious
4. Not at all anxious

Q10. Assuming that the present society in Japan is classified into the following five classes, where do you think you belong?

1. Top
2. Top of the middle
3. Bottom of the middle
4. Top of the bottom
5. Bottom of the bottom

Q11. Are you satisfied with the current healthcare system in general?

1. Highly satisfied
2. Satisfied
3. Partly dissatisfied
4. Dissatisfied

Q12. During the past 4 weeks, have you ever bought or tried to the following treatments or products? (Please circle all that apply)

1. Chinese (kampo) medicine
2. Acupuncture
3. Massage
4. Chiropractic therapy
5. Aromatherapy
6. Supplements (vitamins, nutritional supplements, etc.)
7. Nutritional /nourishing drinks
8. Health promoting tools
9. Qigong
10. Other
11. Have never

Q13. Do you have a paying job now?

1. Yes
2. Yes (temporary left from a job).
3. No (unemployed).
4. No (retired).
5. No (housewife).
6. No (students).
7. No (other).

Q14. Which one of the following categories best describes your job?

1. Executive of a company or a corporation
2. Regular employee
3. Part time temporary worker
4. Dispatched worker from temporary personnel agency
5. Contract or non-regular employee
6. Self-employed or freelance
7. Family worker
8. Doing piece work at home
9. Other

Q15. What is the last school you attended (or are attending now)?

1. Junior high school
2. High school
3. Junior college / College of technology
4. Vocational school
5. University
6. Graduate school
7. Other

Q16. Which one of the following best describes your annual household income last year? Please answer the income before deducting taxes. Include income not only from your jobs, but also from all other sources such as stock shares, pensions, and real estate.

1. None
2. Less than 1 million yen
3. 1 – 2 million yen
4. 2 – 3 million yen
5. 3 – 4 million yen
6. 4 – 5 million yen
7. 5 – 6 million yen
8. 6 – 7 million yen
9. 7 – 8 million yen
10. 8 – 9 million yen
11. 9 – 10 million yen
12. 10 – 12 million yen
13. 12 – 14 million yen
14. 14 – 16 million yen
15. 16 – 19 million yen
16. 19 – 23 million yen
17. 23 million yen or over

Q17. Please enter the number of your household members. (Enter the specific numbers.)

|  |  |
| --- | --- |

Q18. Are you married?

1. Married
2. Unmarried
3. Divorce
4. Widow

| 健康と暮らしに関する意識調査 |
| --- |

**問１－１**　性別

| **１** | 男性 | **２** | 女性 |
| --- | --- | --- | --- |

**問１－１**　年齢を記入してください。

| 現在 |  |  | 歳 |
| --- | --- | --- | --- |

**問２**　一般的に言って、現在のあなたの健康状態はいかがですか。

| **１** | **２** | **３** | **４** | **５** |
| --- | --- | --- | --- | --- |
| とてもよい | まあよい | ふつう | あまり良くない | 悪い |

**問３**　あなたは現在、ご自分の健康に不安を感じることがありますか。

| **１**　いつも  不安を感じる | **２**　ときどき  不安を感じる | **３**　あまり  不安を感じない | **４**　まったく  不安を感じない |
| --- | --- | --- | --- |

**問４**　あなたは現在、診療所や病院で定期的に診てもらっている病気がありますか。

| **１** | **２** |
| --- | --- |
| ある | ない |

**問５－１**　全体的にみて、過去１ヵ月間のあなたの健康状態はいかがでしたか。

| **１** | **２** | **３** | **４** | **５** | **６** |
| --- | --- | --- | --- | --- | --- |
| 最高に  良い | とても  良い | 良い | あまり  良くない | 良くない | ぜんぜん  良くない |

**問５－２**　過去１ヵ月間に、体を使う日常活動（歩いたり階段を昇ったりなど）をすることが身体的な理由でどのくらいげられましたか。

| **１** | **２** | **３** | **４** | **５** |
| --- | --- | --- | --- | --- |
| ぜんぜん  げられなかった | わずかに  げられた | 少し  げられた | かなり  げられた | 体を使う日常活動ができなかった |

**問５－３**　過去１ヵ月間に、いつもの仕事（家事も含みます）をすることが、身体的な理由でどのくらいげられましたか。

| **１** | **２** | **３** | **４** | **５** |
| --- | --- | --- | --- | --- |
| ぜんぜん  げられなかった | わずかに  げられた | 少し  げられた | かなり  げられた | いつもの仕事が  できなかった |

**問５－４**　過去１ヵ月間に、体の痛みはどのくらいありましたか。

| **１** | **２** | **３** | **４** | **５** | **６** |
| --- | --- | --- | --- | --- | --- |
| ぜんぜん  なかった | かすかな  痛み | 軽い痛み | 中くらいの  痛み | 強い痛み | 非常に  激しい痛み |

**問５－５**　過去１ヵ月間、どのくらい元気でしたか。

| **１** | **２** | **３** | **４** | **５** |
| --- | --- | --- | --- | --- |
| 非常に  元気だった | かなり  元気だった | 少し  元気だった | わずかに  元気だった | ぜんぜん  元気でなかった |

**問５－６**　過去１ヵ月間に、家族や友人とのふだんのつきあいが、身体的あるいは心理的な理由で、

どのくらいげられましたか。

| **１** | **２** | **３** | **４** | **５** |
| --- | --- | --- | --- | --- |
| ぜんぜん  げられなかった | わずかに  げられた | 少し  げられた | かなり  げられた | つきあいが  できなかった |

**問５－７**　過去１ヵ月間に、心理的な問題（不安を感じたり、気分が落ち込んだり、イライラしたり）に、どのくらい悩まされましたか。

| **１** | **２** | **３** | **４** | **５** |
| --- | --- | --- | --- | --- |
| ぜんぜん  悩まされなかった | わずかに  悩まされた | 少し  悩まされた | かなり  悩まされた | 非常に  悩まされた |

**問５－８**　過去１ヵ月間に、日常行う活動（仕事、学校、家事などのふだんの行動）が、心理的な

理由で、どのくらいげられましたか。

| **１** | **２** | **３** | **４** | **５** |
| --- | --- | --- | --- | --- |
| ぜんぜん  げられなかった | わずかに  げられた | 少し  げられた | かなり  げられた | 日常行う活動が  できなかった |

**問７**　あなたは、現在の生活全般に満足していますか、それとも満足していませんか。

| **１** | **２** | **３** | **４** | **５** |
| --- | --- | --- | --- | --- |
| 満足している | どちらかといえば  満足している | どちらとも  いえない | どちらかといえば  満足していない | 満足していない |

**問８**　あなたは、将来の自分の暮らしに希望がありますか、それとも希望がありませんか。

| **１**　大いに  希望がある | **２**　希望が  ある | **３**　どちらとも  いえない | **４**　あまり  希望がない | **５**　まったく  希望がない |
| --- | --- | --- | --- | --- |

**問９**　今後の収入や資産の見通しについて、あなたは不安を感じますか、それとも不安を感じませんか。

| **１**　とても  不安である | **２**　ある程度  不安である | **３**　あまり  不安ではない | **４**　まったく  不安ではない |
| --- | --- | --- | --- |

**問10**　かりに現在の日本の社会全体を、以下の５つの層にわけるとすれば、あなた自身は、どれに入ると思いますか。

| **１**　上 | **２**　中の上 | **３**　中の下 | **４**　下の上 | **５**　下の下 |
| --- | --- | --- | --- | --- |

**問11**　あなたは、現在の医療制度全般について満足していますか、それとも満足していませんか。

| **１**　非常に  　　満足している | **２**　まあ  満足している | **３**　あまり  満足していない | **４**　まったく  満足していない |
| --- | --- | --- | --- |

**問12**　次の中で、あなたが過去１ヶ月で購入したり、試したことのあるものはありますか。あてはまるものすべてに○をつけてください。

| **１** | 漢方（保険適用されていないもの） | **６** | サプリメント（ビタミン剤・栄養補助食品など） |
| --- | --- | --- | --- |
| **２** | 鍼灸 | **７** | 栄養ドリンク・滋養強壮剤 |
| **３** | あんま・マッサージ・指圧 | **８** | 健康器具・機械 |
| **４** | カイロプラティック・整体 | **９** | 気功 |
| **５** | アロマセラピー | **10** | その他 |
|  |  | **11** | 購入したり、試したものはない |

**問13**　あなたは、現在、収入をともなうお仕事についていますか（学生アルバイトは除きます）。

| **１** | **２** | **３** | **４** | **５** | **６** | **７** |
| --- | --- | --- | --- | --- | --- | --- |
| 仕事に  ついている | 休職中(仕事から離れて  いるが戻ることになって  いる状態) | 失業中 | 定年などで  仕事をやめた | 主に家事を  している | 学生 | その他の  無職 |

**問14**　あなたご自身の従業上の地位は、大きく分けて、この中のどれにあたりますか。

| **１** | 経営者・役員 | **４** | 派遣社員 | **７** | 家族従業者 |
| --- | --- | --- | --- | --- | --- |
| **２** | 常時雇用の一般従業員 | **５** | 契約社員、嘱託 | **８** | 内職 |
| **３** | 臨時雇用、パート、アルバイト | **６** | 自営業主、自由業者 | **９** | その他 |

**問15**　あなたが最後に行かれた（または現在行っている）学校は次のどれにあたりますか。中退も卒業と同じ扱いでお答えください。

| **１** | 中学校 | **５** | 大　学 |
| --- | --- | --- | --- |
| **２** | 高　校 | **６** | 大学院 |
| **３** | 短大・高専 | **７** | その他 |
| **４** | 専修学校（高卒後） |  |  |

**問16**　昨年１年間の収入は、税込みでいくらくらいでしょうか。家族全体の収入について、当てはまるもの選んでください。仕送り・年金・生活保護などをうけている場合には、その額も含めてお答えください。

| **１** | なし | **７** | 500～600万円未満 | **13** | 1200～1400万円未満 |
| --- | --- | --- | --- | --- | --- |
| **２** | 100万円未満 | **８** | 600～700万円未満 | **14** | 1400～1600万円未満 |
| **３** | 100～200万円未満 | **９** | 700～800万円未満 | **15** | 1600～1900万円未満 |
| **４** | 200～300万円未満 | **10** | 800～900万円未満 | **16** | 1900～2300万円未満 |
| **５** | 300～400万円未満 | **11** | 900～1000万円未満 | **17** | 2300万円以上 |
| **６** | 400～500万円未満 | **12** | 1000～1200万円未満 |  |  |

**問17**　現在、同居されているご家族の方は、あなたを含めて何人ですか。

|  | 人 |
| --- | --- |

**問18**　あなたは現在、ご結婚されていますか。

| **１** | 既婚 | **２** | 未婚 | **３** | 離別 | **４** | 死別 |
| --- | --- | --- | --- | --- | --- | --- | --- |
